# Combined video-assisted thoracoscopy surgery and posterior midline incision for *en bloc* resection of non-small-cell lung cancer invading the spine

**DOI:** 10.1093/icvts/ivab215

**Published:** 2021-07-30

**Authors:** Kheira Hireche, Mathieu Moqaddam, Nicolas Lonjon, Charles Marty-Ané, Laurence Solovei, Baris Ata Ozdemir, Ludovic Canaud, Pierre Alric

**Affiliations:** 1 Department of Thoracic and Vascular Surgery, Arnaud de Villeneuve Hospital, Montpellier, France; 2 Department of Neurosurgery, Gui de Chauliac Hospital, Montpellier, France; 3 PhyMedExp, University of Montpellier, INSERM, CNRS, Montpellier, France; 4 University of Bristol, Bristol, UK

**Keywords:** NSCLC, Lobectomy, VATS, Spine

## Abstract

**OBJECTIVES:**

This article aims to evaluate the feasibility and safety of a hybrid video-assisted thoracic surgery (VATS) approach to achieve *en bloc* lobectomy and spinal resection for non-small-cell lung cancer (NSCLC).

**METHODS:**

Between October 2015 and November 2020, 10 patients underwent VATS anatomical lobectomy and *en bloc* chest wall and spinal resection through a limited posterior midline incision as a single operation for T4 (vertebral involvement) lung cancer. Nine patients had Pancoast syndrome without vascular involvement and 1 patient had NSCLC of the right lower lobe with invasion of T9 and T10.

**RESULTS:**

There were 5 men and 5 women. The mean age was 61 years (range: 47–74 years). Induction treatment was administered to 9 patients (90%). The average operative time was 315.5 min (range: 250–375 min). The average blood loss was 665 ml (range: 100–2500 ml). Spinal resection was hemivertebrectomy in 6 patients and wedge corpectomy in 4 patients. Complete resection (R0) was achieved in all patients. The average hospitalization stay was 14 days (range: 6–50 days). There was no in-hospital mortality. The mean follow-up was 32.3 months (range: 6–66 months). Six patients (60%) are alive without recurrence.

**CONCLUSIONS:**

VATS is feasible and safe to achieve *en bloc* resection of NSCLC inviding the spine without compromising oncological efficacy. Further experience and longer follow-up are needed to determine if this approach provides any advantages over thoracotomy.

**Figure:**
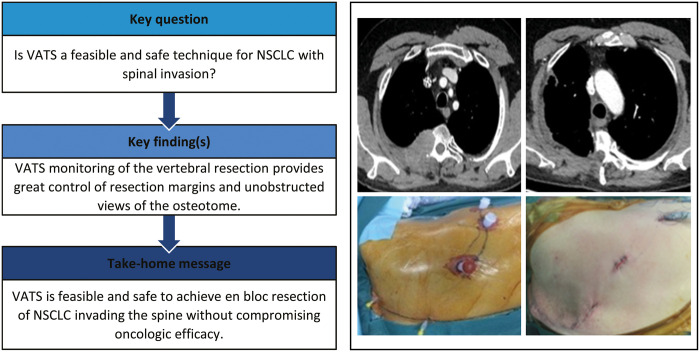

## INTRODUCTION

Given anatomical proximity, pulmonary sulcus non-small-cell lung cancer (NSCLC) and costovertebral gutter tumours are prone to spinal invasion. Although challenging, *en* *bloc* extra-tumoural resection is the only oncologically acceptable end point. From the first description of successful *en* *bloc* resection in Pancoast tumours in 1953 by Chardack and Maccallum [1] to the first report of a successful total vertebrectomy for *en* *bloc* resection in 1996 by Grunenwald *et al.* [2], several limitations to surgical resection have been overcome. Multiple approaches have been described for complex surgical resections [3–5].

The posterolateral thoracotomy reported by Shaw *et al.* in 1961 [3] is the most widely adopted surgical technique for local spinal invasion of NSCLC. This surgical approach is extensive and involves substantial collateral tissue damage leading to increased postoperative pain and respiratory complications. Video-assisted thoracic surgery (VATS) is gaining momentum with the publication of increasing numbers of studies and the development of clinical guidelines, now often considered first line in the treatment of early-stage lung cancer [6].

High-volume centres have evaluated and validated the effectiveness of VATS for locally advanced NSCLC extending to the chest wall, resulting in considerably increased acceptance [7, 8]. Studies on the feasibility and safety of VATS lobectomy with spine resection are however insufficient. The purpose of this study was to test hybrid minimally invasive approach combining VATS lobectomy with spinal resection for NSCLC with vertebral invasion.

## MATERIALS AND METHODS

### Patient data

From October 2015 to November 2020, 10 patients underwent concomitant *en* *bloc* VATS lobectomy and spinal resection through a limited posterior midline incision for T4 NSCLC with curative intent. During the same period, 920 anatomical VATS lobectomies were performed in the study institution. Inclusion criteria were preoperative histologically proven NSCLC with radiologically demonstrated spinal invasion, without gross multistation disease prior to therapy. Patients with subclavian vessel invasion, spinal canal involvement or spinal invasion requiring total vertebrectomy or hemivertebrectomy on >3 levels and patients with metastatic disease were excluded.

Preoperative computed tomography (CT)-guided biopsy and histology was used to confirm the diagnosis of NSCLC. The preoperative workup included pulmonary function tests, bronchoscopy, CT of the chest, abdomen and pelvis, positron emission tomography and magnetic resonance imaging (MRI) of the brain and spine. Endobronchial ultrasonography was used for invasive nodal staging prior to induction therapy in all patients.

All patients but 1 underwent neo-adjuvant therapy. Seven patients received concurrent chemoradiotherapy comprising 3 cycles of cisplatin and vinorelbine and fractionated radiotherapy to a total dose of 45 Gr. Two patients were treated with chemotherapy using cisplatin and vinorelbine (3 cycles). One patient with tumour-associated haemoptysis underwent surgery without induction therapy.

**Figure 1:**
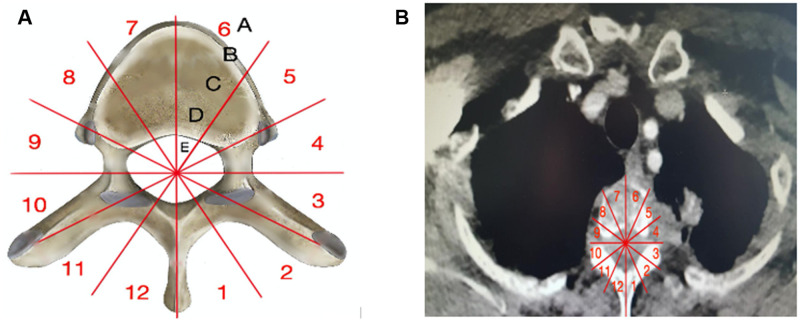
(**A**) Modified Weinstein, Boriani and Biagini surgical staging system by consensus of the Spine Oncology Study Group. (**B**) Preoperative computed tomography scans: tumour located in zones 3–5 with intervertebral foramina invasion.

Three weeks after the completion of induction treatment, all patients underwent repeat CT and spinal MRI. Preoperative spinal invasion and planed resection were assessed on post-induction MRI using the Weinstein, Boriani and Biagini surgical staging system [9] that divided the vertebra into 12 radiating zones numbered 1–12 clockwise on the transverse plane and into 5 layers A to E from prevertebral to dural involvement (Fig. 1).

To exclude residual active mediastinal disease after induction therapy, repeat endobronchial ultrasonography or mediastinoscopy staging was performed in patients with N2 disease. Patients with persistent N2 disease after induction therapy were not offered resection. Surgery was scheduled 4 –6 weeks after the completion of induction treatment.

### Surgical technique

All surgical procedures were performed under general anaesthesia, with the use of single-lung ventilation through a double-lumen endotracheal tube. The patients were placed in the lateral decubitus position. Three video access ports through 12-mm skin incisions were established, and the pleural space was entered.

**Figure 2:**
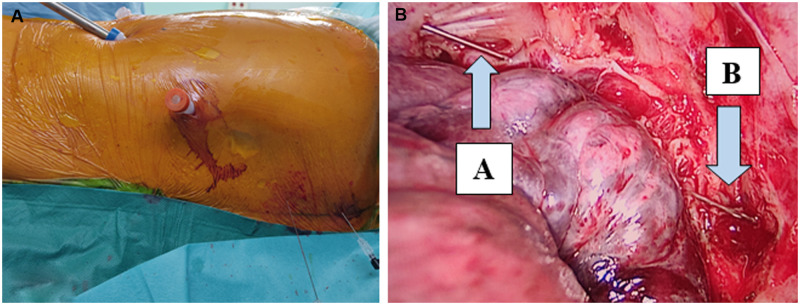
(**A**) Video-assisted thoracic surgery with 3 video access ports. (**B**) Cautirezation of the parietal pleura within 2-cm margin around the tumour. (**A**) Needle at the upper extrimity of the tumour. (**B**) Needle at the lower extrimity.

Thoracoscopic exploration of pleural cavity was performed to exclude macroscopic pleural dissemination and evaluate the relationship of the lung with the adjacent vertebra. A standard VATS lobectomy was performed as the initial step. Adhesions of the lobe to the chest wall did not interfere with the dissection of hilar structures. The pulmonary lobe vein, artery and bronchus were dissected and stapled, followed by fissure disconnection, and lymphadenectomy (stations 5, 6, 7, 8, 9, 10, 11, 4R and 2R). Internal preparation for chest wall resection was performed using a long diathermy hook. The parietal pleura was cauterized 2 cm from the tumour margin to achieve histological clearance, and then 2 peridural needles were pinned into the chest wall from outside to inside under VATS guidance at the upper and lower pole of the tumour, guiding the extent of the spinal resection and the posterior midline incision (Fig. 2). The pulmonary lobe was left in place attached *en bloc* to the invaded spine, according to oncological principles.

**Figure 3:**
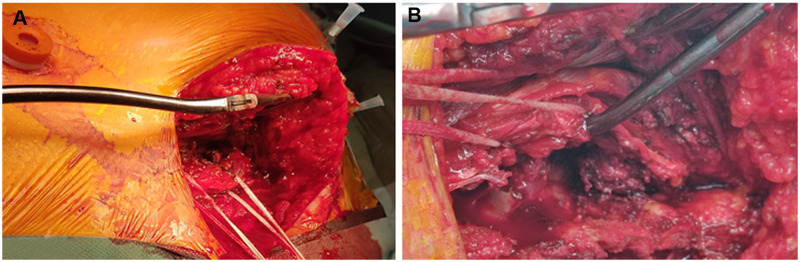
(**A**) Needle trageted limited posterior approach. Trapezius and rhomboid muscles were cut to expose vertebrae and ribs. (**B**) Paravertebral muscles are split alongside fibres without transection.

Next, the spinal resection was permormed by the spine surgeon through a needle targeted 15–20 cm ‘L’ posterior midline skin incision. A muscle-sparing technique splitting the paravertebral muscles was used to expose the vertebrae (Fig. 3). The trapezius and rhomboid muscles were cut to expose the posterior arches of the ribs; the latissimus dorsi muscle was always preserved. The scapula was retracted without the need of muscular release. If the posterior and anterior vertebral elements according to the Boriani staging system (intervertebral foramina, pedicles and vertebral body) were involved, a hemivertebrectomy was mandatory to provide adequate clearance. In these patients, the tumour was isolated from the uninvolved vertebra by performing a unilateral laminectomy preserving the spinous process. The dura and nerve roots were then exposed. Invaded nerve roots were dissected, clipped, and divided to prevent cerebrospinal fluid leakage. The osteotomy of the vertebral body was performed under VATS guidance from posterior to anterior medial to the ipsilateral pedicle, allowing the removal of facets and pedicle *en* *bloc* with the tumour. Osteotomy of the involved vertebral bodies was performed with osteotomes and followed by the section of the anterior longitudinal ligament under a direct visual video-assisted control. A safety margin was respected to avoid any encroachment of the tumour. In patients where only the transverse process or costovertebral groove was involved, a wedge corpectomy was necessary to achieve an extended and complete R0 resection. No patient underwent spinal instrumentation.

**Figure 4:**
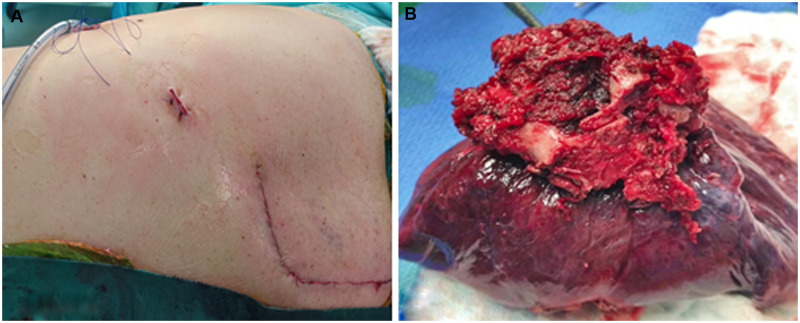
(**A**) Postoperative scars. (**B**) The specimen was removed *en bloc*.

Finally, the posterior arches of the involved ribs and related intercostal muscles were resected without the need for additional thoracotomy and rib spreading. The specimen was removed *en* *bloc* with the involved vertebrae, tumour, and ribs through the posterior incision (Fig. 4). After resection, an epidural catheter was placed under direct visual control in case of hemivertebectomies. In all the cases, with the exception of 1 patient who had a lower lobectomy, the scapula covered the chest wall defect without the need for reconstruction. One intercostal drain was placed through the VATS port incision and the posterior midline incision was closed.

Pain was evaluated using a 1–10 visual analogue scale score. For the first 48 postoperative hours, the pain control was achieved by continuous thoracic epidural analgesia, intravenous non-steroidal anti-inflammatory drugs (Profenid) and intravenous paracetamol. Long-term pain control was then managed by the combination of trans-cutaneous narcotics (durogesic), oral narcotics (oxyquentin), oral tramadol and oral paracetamol. All patients were evaluated 1 month after surgery for the pain and type of analgesic administered.

Surgical technique of combined *en* *bloc* VATS left upper lobectomy and spinal resection is summarized in Video 1.

## RESULTS

All related information is summarized in Table 1.

**Table 1:** Patients and surgical characteristics

*N*	Age	cTNM	Induction therapy	WBB sector	Type of spine resection	Rib resection	Length of surgery (min)	Blood loss (ml)	Transfusion (CGR)	Hospital stay (day)	Complications	Histology	pTNM	Tumours margin	Recurrence	Data follow-up
1	57	T3N0M0	C	10–9	WC T1, T2, T3	I, II, III rib	300	400	2	50	Chylothorax, peritonitis	Adenocarcinoma	T3N2M0	R0	Local	Alive at 34 months
2	47	T4N0M0	CR	8–10	Hemivertebrectomy T2, T3, T4	II, III, IV, V rib	330	2500	4	14	Pneumonia	Adenocarcinoma	T4N0M0	R0	No	Alive at 66 months
3	65	T3N2M0	CR	8–10	WC T1, T2, T3	I, II, III rib	320	300	2	9	No	Adenosquamous	T3N1M0	R0	Brain adrenal metastasis	Death at 13 months
4	74	T4N1M0	CR	3–5	WC T2, T3	I, II, III, IV rib	250	100	0	11	Pleural effusion	Squamous cell	T3N0M0	R0	No	Alive at 59 month
5	50	T4N0M0	CR	8–10	Hemivertebrectomy T2, T3	II, III rib	300	1600	4	9	No	Poorly differentiated	T0N0M0	R0	No	Alive at 41 months
6	69	T4N0M0	None	7–9	Hemivertebrectomy T2, T3, T4 + TP T1, T5	I, II, III, IV, V rib	330	600	3	12	Air leakage	Adenocarcinoma	T4N0M0	R0	Brain bone metastasis	Death at 15 months
7	66	T4N0M0	C	8–10	Hemivertebrectomy T9, T10	VIII, IX, X, XI rib	375	500	2	9	No	Adenocarcinoma	T4N2M0	R0	Local	Alive at 40 months
8	59	T4N0M0	CR	8–10	Hemivertebrectomy T2, T3, T4	II, III, IV rib	330	300	0	12	Pleural effusion	Adenocarcinoma	T0N0M0	R0	No	Alive at 31 months
9	69	T4N0M0	CR	8–10	Hemivertebrectomy T1, T2, T3	I, II, III rib	300	200	0	8	No	Squamous cell	T3N0M0	R0	No	Alive at 18 months
10	54	T4N0M0	CR	3–5	WC T2, T3	II, III rib	320	150	0	6	No	Adenocarcinoma	T0N0M0	R0	No	Alive at 6 months

C: chemotherapy; CR: chemoradiotherapy; TP: Transverse process; WBB: Weinstein, Boriani and Biagini; WC: wedge corpectomy.

There were 5 males and 5 females. Ages ranged from 47 to 74 years (average 61 years). Tumour was in the right upper lobe in 7, the left upper lobe in 2 and the right lower lobe in the final patient.

### Histology and tumour margin

The histological distribution was: 6 adenocarcinoma, 2 squamous cell carcinoma, 1 adenosquamous carcinoma and 1 poorly differentiated NSCLC. Pathological stages in resected specimens were T0N0 in 3 (30%), T3N0 in 3 (30%), T4N0 in 1 (20%), T3N1 in 1 (10%), T3N2 in 1 (10%) and T4N2 in 1 (10%). The median tumour size was 47 mm (38–60 mm). Microscopic analysis and measurement of the resection margin were systematically performed; complete microscopic resection (R0) was achieved in all patients.

### Type of resection

All tumours were resected *en* *bloc*. The number of vertebral levels resected ranged from 2 to 5 (average, 2.8 levels). Spinal resection was performed at 2 levels in 4 patients (T2–T3 = 3, T9–T10 = 1), at 3 levels in 5 patients (T1–T3 = 3, T2–T4 = 2) and at 5 levels in 1 patient (T1–T5). In this latter, patient hemivertebrectomy was done from T2–T4 and wedge corpectomy with TP resection at T1 and T5. Thoracic wall resection was performed in all patients. The number of resected ribs ranges from 2 to 5 (average 3.4 ribs) including 2 ribs in 2 patient, 3 ribs in 4 patients, 4 ribs in 3 patients and 5 ribs in 1 patient.

### Surgical outcomes and postoperative complications

No patient needed conversion to thoracotomy. The mean operative duration was 315.5 min (range: 250–375 min). The mean blood loss was 665 ml (range: 100–2500 ml). The mean number of packed red blood cells transfused during the procedure was 1.7 (range: 0–4 packs). The mean hospital stay length was 14 days (range: 6–50 days).

In the postoperative period, complications occurred in 5 patients, prolonged air leakage in 1 patient, pleural effusion in 2 patients and pulmonary infection successfully treated by antibiotics and physiotherapy in 1 patient. Another 57-year-old male patient had a chelothorax and peritonitis following percutaneous gastrostomy tube insertion successfully managed by surgery. This patient's hospital stay was 50 days. There were no perioperative deaths.

One month after surgery, 3 patients were still taking oral narcotics, and 3 patients received oral tramadol and paracetamol. The remaining 4 patients had an isolated complaint of occasional low-grade chest pain well controlled by oral paracetamol.

### Follow-up and survival

Follow-up was 32.3 months (range: 6–66 months), with no losses to follow-up. Eight patients (80%) remain alive, among them 6 (60%) recurrence free. Two patients died of distant metastasis at 13 and 15 months, respectively, and one of them (patient number 6) had not undergone neo-adjuvant therapy. Local recurrence was observed in 2 patients. Both were N2 at final staging; 1 of these patients (patient number1) had local and mediastinal lymph node recurrence treated by chemotherapy–immunotherapy and chest wall irradiation. The other (patient number 7) had recurrence at the bronchial stump 7 months after surgery, successfully managed by adjuvant chemotherapy and immunotherapy and then completion pneumonectomy. This patient is alive without recurrence at follow-up.

## DISCUSSION

In the past 2 decades, VATS has become the established standard for treating early-stage NSCLC. The benefits of this approach include expedited recovery with shorter hospital stay, reduced pulmonary complications associated with the reduction in chest wall trauma compared to thoracotomy and better quality of life in the first year after surgery [10–12].

Several authors [13, 14] have reported the feasibility and the safety of Pancoast tumours resection through the trans-manubrial approach of Grunenwald and Spaggiari combined with VATS lobectomy. In the study institution the use of VATS for early-stage NSCLC started in the late 1990s [15]. This experience prompted the study authors to consider using VATS in complex post-chemotherapy, chest wall and spine resections to reduce surgical trauma and facilitate recovery. There are no randomized studies supporting the benefits of VATS in the surgical management of locally advanced NSCLC. However, it is rather expected to believe that the avoidance of musculature division and rib spreading would result in less trauma and decreased pain.

Recently, Caronia *et al.* [14] and Nun *et al.* [16] published retrospective comparative studies reporting the use of video assistance in tumours of the thoracic inlet anterior compartment demonstrating shorter hospital stay, reduced pain and analgesic consumption following the hybrid approach compared to thoracotomy.

In 2013, Stoker *et al.* [17] reported the use of VATS for *en* *bloc* lobectomy and spinal resection in 4 patients. In this study, spinal resection and instrumentation were performed through posterior midline incision in prone position without video guidance. Unlike stoker *et al.*, all patients in this study were placed in the lateral decubitus position during the whole surgical process, which is time sparing. More importantly, spinal resection was performed under video assistance. With this manoeuvre, safe resection borders can be prepared easily using cautery around the tumour and make the rib resection easier and more precise. VATS allowed monitoring of the vertebral resection providing greater control of resection margins and unobstructed views of osteotome position relative to surrounding structures, improving therefore the safety of the procedure.

There is no consensus on spinal instrumentation after partial vertebrectomy for NSCLC. However, most authors perform at least anterior or posterior instrumentation, or even both [18–20]. Bolton *et al.* performed instrumentation only in case of total vertebrectomy [21]. The operative morbidity and neurological complications associated with these procedures is not insignificant. Compression, fracture and spinal device dislocation are well-known complications that can lead to emergent or delayed surgical revisions [18–20]. In the absence of validated criteria to justify systematic spinal instrumentation and in view of its inherent risks, in the present study, spinal instrumentation was not performed prima facie. All patients are then assessed clinically and radiologically by spinal surgeons; selective spine stabilization is performed for patient with the high risk of deformity in a manner as previously described [22].

The incidence of respiratory complications for *en* *bloc* resection of pulmonary sulcus tumours invading the spine varies from 27% [19] to 41% [18]. It seems relevant to try to reduce these complications since the primary cause of mortality in combined chest wall and lung resection is respiratory insufficiency. In the present series, only 1 patient had pneumonia successfully managed by antibiotics and aggressive physiotherapy. The lower trauma resulting from the VATS approach improves pain control and allows the patients to sustain more vigorous physiotherapy during the postoperative period than patients operated on by traditional thoracotomy. In their paper, Caronia *et al.* [14] reported more respiratory complications and more frequent bronchoscopic aspiration in the thoracotomy group. Likewise, Nun *et al.* [16] reported 4 times lower lung infections in the VATS group.

Another advantage of this approach is that video assistance allowed reduction in skin incision size. It is well established that radiation therapy increases the likelihood of operative complications [23]. In his study about surgery for metastatic disease of the spine, Wise *et al.* [24] reported 11% of surgical site infections all in patients who received preoperative irradiation. Likewise, several series of *en* *bloc* spine resection of NSCLC reported serious deep surgical wound infections [17, 18, 20]. The authors believe that tissue damages related to radiotherapy added to the injuries generated by wide exposure of open thoracotomy make these patients more vulnerable to surgical site complications. In fact, reducing wound magnitude may be particularly beneficial after external radiation.

Multimodality therapy has become the treatment of choice of those tumours. Following chemoradiotherapy, Rusch *et al.* [25] reported a 44% 5-year survival rate, and Anraku *et al.* [26] reported complete pathological response as high as 48%. The results reported here after induction chemoradiotherapy are very encouraging. Complete pathological response was observed in 3 patients (30%) and >90% in 3 others. Inasmuch as complete resection (R0) is the most widely reported predictive factor for long-term survival [27, 28] and frozen-section histology is not feasible on bone, partial corpectomy was deliberately selected to obtain clear margins for tumours with costo-vertebral groove invasion documented on postinduction radiographic findings. With this aggressive surgical procedure performed via a mini-invasive approach complete resection with free margin was achieved in all patients, 80% overall survival rate and 60% recurrence free survival at the mean follow-up of 32.3 months. These results are similar to other series [18–20, 25, 26].

### Limitations

This study is limited by the modest number of patients enrolled and by its single-centre retrospective nonrandomized nature. The insufficient follow-up duration and the lack of case-matched comparison limit our ability to draw solid conclusions about the potential advantages of this approach over thoracotomy. Thus, our results should be interpreted with caution.

## CONCLUSION

The results of this study suggest that *en bloc* VATS lobectomy and spine resection are feasible and safe. The VATS approach affords better appreciation of the extent of spine and chest wall resection whilst also reducing the additional morbidity of a large thoracotomy.

However, more studies ideally randomized but more realistically in the form of multicentre registries are required to define the appropriate role of hybrid VATS approach in the management of NSCLC with spine invasion.

## Funding

No funding was provided.

##  

**Conflict of interest:** none declared.

## Author contributions

**Kheira Hireche:** Conceptualization; Formal analysis; Investigation; Methodology; Writing—original draft. **Mathieu Moqaddam:** Data curation. **Nicolas Lonjon:** Supervision. **Charles Marty-Ané:** Supervision. **Laurence Solovei:** Investigation. **Baris Ata Ozdemir:** Writing—review & editing. **Ludovic Canaud:** Visualization. **Pierre Alric:** Visualization.

## Reviewer information

Interactive CardioVascular and Thoracic Surgery thanks Domenico Galetta, Thomas F. Molnar and Matthieu Thumerel for their contribution to the peer review process of this article.

## ABBREVIATIONS

CT: Computed tomography
MRI: Magnetic resonance imaging
NSCLC: Non-small-cell lung cancer
VATS: Video-assisted thoracic surgery
